# Identification of Ethical Issues and Practice Recommendations Regarding the Use of Robotic Coaching Solutions for Older Adults: Narrative Review

**DOI:** 10.2196/48126

**Published:** 2024-06-18

**Authors:** Cécilia Palmier, Anne-Sophie Rigaud, Toshimi Ogawa, Rainer Wieching, Sébastien Dacunha, Federico Barbarossa, Vera Stara, Roberta Bevilacqua, Maribel Pino

**Affiliations:** 1 Maladie d’Alzheimer Université de Paris Paris France; 2 Service de Gériatrie 1 & 2 Hôpital Broca Assistance Publique - Hôpitaux de Paris Paris France; 3 Smart-Aging Research Center Tohoku University Sendai Japan; 4 Institute for New Media & Information Systems University of Siegen Siegen Germany; 5 Scientific Direction Istituto Nazionale di Ricovero e Cura per Anziani Ancona Italy

**Keywords:** robotic coaching solutions, ethical issues, ethical recommendations, older adults, geriatrics, guidelines

## Abstract

**Background:**

Technological advances in robotics, artificial intelligence, cognitive algorithms, and internet-based coaches have contributed to the development of devices capable of responding to some of the challenges resulting from demographic aging. Numerous studies have explored the use of robotic coaching solutions (RCSs) for supporting healthy behaviors in older adults and have shown their benefits regarding the quality of life and functional independence of older adults at home. However, the use of RCSs by individuals who are potentially vulnerable raises many ethical questions. Establishing an ethical framework to guide the development, use, and evaluation practices regarding RCSs for older adults seems highly pertinent.

**Objective:**

The objective of this paper was to highlight the ethical issues related to the use of RCSs for health care purposes among older adults and draft recommendations for researchers and health care professionals interested in using RCSs for older adults.

**Methods:**

We conducted a narrative review of the literature to identify publications including an analysis of the ethical dimension and recommendations regarding the use of RCSs for older adults. We used a qualitative analysis methodology inspired by a Health Technology Assessment model. We included all article types such as theoretical papers, research studies, and reviews dealing with ethical issues or recommendations for the implementation of these RCSs in a general population, particularly among older adults, in the health care sector and published after 2011 in either English or French. The review was performed between August and December 2021 using the PubMed, CINAHL, Embase, Scopus, Web of Science, IEEE Explore, SpringerLink, and PsycINFO databases. Selected publications were analyzed using the European Network of Health Technology Assessment Core Model (version 3.0) around 5 ethical topics: benefit-harm balance, autonomy, privacy, justice and equity, and legislation.

**Results:**

In the 25 publications analyzed, the most cited ethical concerns were the risk of accidents, lack of reliability, loss of control, risk of deception, risk of social isolation, data confidentiality, and liability in case of safety problems. Recommendations included collecting the opinion of target users, collecting their consent, and training professionals in the use of RCSs. Proper data management, anonymization, and encryption appeared to be essential to protect RCS users’ personal data.

**Conclusions:**

Our analysis supports the interest in using RCSs for older adults because of their potential contribution to individuals’ quality of life and well-being. This analysis highlights many ethical issues linked to the use of RCSs for health-related goals. Future studies should consider the organizational consequences of the implementation of RCSs and the influence of cultural and socioeconomic specificities of the context of experimentation. We suggest implementing a scalable ethical and regulatory framework to accompany the development and implementation of RCSs for various aspects related to the technology, individual, or legal aspects.

## Introduction

### Challenges Associated to Population Aging

Technological and medical advances have led to a demographic shift in the population, with the number of older adults constantly increasing. According to the United Nations [1], older adults (aged 60-65 years) will represent 16% of the world’s population in 2050. In addition, life expectancy is increasing, from 64.2 years in 1990 to 72.6 years in 2019, and is expected to reach 77.1 years in 2050 [1]. However, there is a wide diversity of health conditions among older adults. The health status of older adults is dependent on multiple factors, including nonmodifiable genetic factors and environmental factors, such as lifestyle [2]. Thus, older adults represent a very heterogeneous population with multiple and diverse needs and desires. With advancing age, the loss of functional independence; frailty; and other health diseases such as cardiovascular problems, cancers, osteoarthritis, osteoporosis, or major neurocognitive disorders may appear [3-5]. Among age-related conditions, major neurocognitive disorders (eg, Alzheimer disease) receive particular attention due to the increasing prevalence of these diseases [6].

The aging population is not only a public health issue but also a socioeconomic one. To face this challenge, it is important to develop preventive measures to support active and healthy aging and to preserve the independent functioning and quality of life of older adults. The adoption of healthy behaviors can help prevent or delay the onset of pathologies or treat them if detected early [7].

### The Use of Technologies for Older Adults

Preventive health measures can be supported through new technologies, such as robotic coaching solutions (RCSs) that promote healthy aging among older adults [8,9]. RCSs have been defined as personalized systems that continuously monitor the activities and environment of the user and provide them with timely health-related advice and interventions [10-12]. These systems can help users define and achieve different health-oriented goals [12].

RCSs may encompass artificial intelligence (AI) technologies that can analyze user data, personalize coaching programs, and adapt recommendations based on each individual’s needs [1,13-19]. RCSs can involve robots equipped with sensors such as cameras, microphones, or motion sensors to collect real-time data about the user, AI, and programming that enables their interaction with users [20,21]. These technologies are often equipped with voice and visual recognition and learning capabilities [20,21]. They can benefit from advanced natural language processing techniques, which allow for understanding of the user’s input, facilitating natural and effective communication [22]. RCSs can offer guidance, support, and feedback based on preprogrammed information or real-time data analysis. These data can inform coaching strategies and allow RCSs to provide users with relevant feedback [8].

RCSs can also encompass a virtual agent, which refers to a computer program or an AI system that interacts with users in a manner that simulates human conversation [14,18,23]. A virtual agent is an animated character capable of adopting a social behavior mimicking that of humans to encourage the users to make changes in their habits [14]. Virtual agents might take the form of a chatbot, voice assistant, or other AI-driven communication system [14]. Biometric monitoring devices to track physiological data such as heart rate, sleep patterns, or stress levels can also be included in RCSs [8,20,21]. These data can contribute to the configuration of personalized coaching plans. RCSs can also encompass advanced data analytics that can process large data sets generated by users’ interactions and behaviors. This functionality helps in identifying patterns, trends, and areas for improvement in coaching strategies [24]. Integrating Internet-of-Things devices in RCSs can provide additional data points about a user’s environment, lifestyle, or habits, thus contributing to a personalized coaching approach [25].

Health-oriented RCSs could enable users to lead a healthy lifestyle, by identifying needs and goals and providing appropriate risk predictions and individualized recommendations [12,26-28]. There are RCSs dedicated to a particular domain, such as physical activity or motor rehabilitation [9,16]. Others may have the objective of promoting independent and healthy aging [29].

Promoting active and healthy aging can allow older adults to maintain their independence and continue to live at home [4,30], which is a wish of many [3]. This intervention could also help to reduce the need for assistance, usually provided by informal caregivers and health professionals [4,19,30-33]. Furthermore, RCSs could lead to a reduction in individual and collective health care expenses [4,32,34] by easing access to health and social care interventions to a wide population, including hard-to-reach (eg, geographically isolated) individuals. However, although the use of health-related RCSs could have many benefits, several ethical issues arise with their development and implementation in human environments [3,35-38].

### An Ethical Framework for the Use of Technologies for Older Adults

For RCSs to contribute to active and healthy aging, it is important that all the stakeholders (engineers, geriatricians, psychologists, etc) involved in their design and implementation refer to an ethical framework [3,38]. It is also important to inform society (politicians and legal experts) about such an extension of technology in people’s lives (private, professional, medicosocial, and commercial context), so that we can create a legal framework for the use of these technologies. An analysis of the way in which ethical and legal dimensions have been addressed by studies, in the field of RCSs for health care, seems useful to support the key actors in their development and implementation. The growing interest in the ethical questions associated with the use of social and assistive robots is evidenced by the volume of literature reviews [3,12,18,31,32,37,39-51] on the topic.

Now, it appears appropriate to systematically examine this body of work, focusing on the ethical analysis, and provide an overview of the literature. Therefore, we performed a review of the literature on RCSs for older adults using the European Network of Health Technology Assessment (EUnetHTA Core Model; version 3.0) model [52] for analysis. This Health Technology Assessment (HTA) model makes it possible to assess the intended and unintended consequences of the use of a specific technology regarding multiple domains (eg, technological, ethical, clinical, and organizational), providing methods and concepts for this analysis [53]. Therefore, HTA is a process that informs decision-making about the introduction of new technologies such as RCSs in health care. It also seems necessary to issue guidelines for the development and implementation of health-oriented RCSs [54].

The objective of this study was to highlight the main ethical questions and corresponding recommendations linked to the use of RCSs for older adults for engineers, researchers, and health professionals in this field. For this purpose, we conducted a narrative literature review using the ethical dimension of the EUnetHTA Core Model to guide the analysis. To the best of our knowledge, such a study has not been conducted so far.

## Methods

### Overview

A thematic analysis of the literature was performed to identify publications that describe RCSs for supporting older adults in health care and prevention and those that address ethical issues and recommendations regarding their development and implementation. The methodology used for the narrative review was inspired by the study by Green et al [55].

### Inclusion and Exclusion Criteria

The review encompassed papers focusing on all populations, with particular attention to older adults. It focused on the concept of RCSs for health, while also incorporating publications discussing other health technologies for older adults if the authors have delved into relevant ethical considerations for their development or implementation.

The context of the review revolved around the use of RCSs (or related technologies), especially for older adults, across diverse living environments such as homes, hospitals, and nursing homes. Publications addressing RCSs and related ethical issues within the health care domain were considered, whereas those focusing solely on technical aspects (eg, AI and deep learning) or those outside the health care domain were excluded.

Various types of publications, including theoretical papers, research studies, and reviews, were included if they offered ethical reflections or recommendations for RCS use in health care. These reflections and recommendations were expected to align with the topics and issues of the ethical dimension of the EUnetHTA Core Model.

All publications, regardless of language (English or French), were eligible if published after 2011. This time frame was chosen considering the technological advancements over the past decade, which may have influenced the evolution of ethical issues and recommendations in the field of remote care systems and related technologies. Textbox 1 summarizes the inclusion and exclusion criteria adopted for the selection of papers in this review.

**Inclusion and exclusion criteria for the selection of publications about ethical issues regarding the use of robotic coaching solutions (RCSs) for older adults.**

**Inclusion criteria**

- Types of participants: all populations
- Interventions or phenomena of interest: RCSs or other technologies used in health care, if ethical issues are discussed
- Context: the use of RCSs in the health care sector
- Paper type: all paper types (theoretical papers, research studies, and reviews) that discuss ethical issues
- Language: English or French
- Date of publication: after 2011

**Exclusion criteria**

- Types of participants: not applicable
- Interventions or phenomena of interest: RCSs or all other types of technology outside the health care sector
- Context: the use of RCSs in non–health care sectors
- Paper type: papers about RCSs and other technologies that are not dealing with ethical issues
- Language: all other languages
- Date of publication: before 2011

### Search Strategy and Study Selection

The review was conducted using the following keywords: “seniors,” “older adults,” “social robots,” “assistive robots,” “assistive technology,” “robots,” “virtual coach,” “e-coaching,” “coaching system,” “coaching device,” “ethics,” and “recommendations.”

The review was performed between August 2021 and December 2021 using the PubMed, CINAHL, Embase, Scopus, Web of Science, IEEE Explore, SpringerLink, and PsycINFO databases.

This search allowed us to find 4928 initial publications. Then, secondary research using references from other articles and the same inclusion criteria was conducted. This search allowed us to find 13 additional papers.

**Figure 1:**
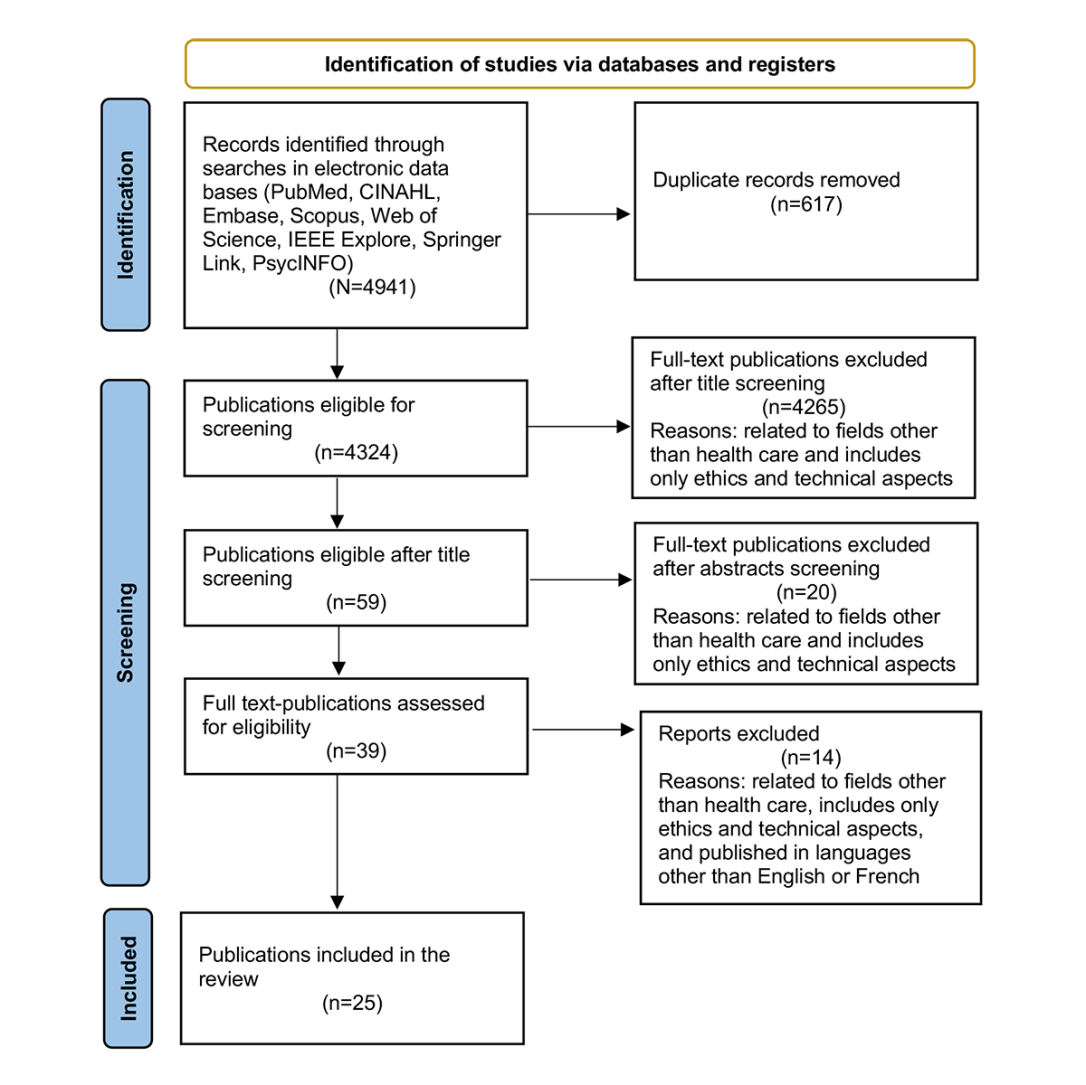
PRISMA (Preferred Reporting Items for Systematic Reviews and Meta-Analyses) flowchart showing a summary of the screening and selection process of publications about ethical issues regarding the use of robotic coaching systems for older adults.

In total, 4943 papers were analyzed. The selection of the final publications was performed after reading the title and abstract first and, then, the full article. This selection process helped us to exclude irrelevant papers and duplicates (Figure 1). In total, 0.51% (25/4943) of the papers were included in our review.

### Data Analysis Criteria

The selected papers were analyzed using the ethical domain of the EUnetHTA Core Model [52]. Proper registration of the use of EUnetHTA Core Model for the purpose of this review was made on the HTA Core Model website [52].

The model was developed for the production and sharing of HTA information, allowing for the support of evidence-based decision-making in health care, but it can also be customized to other research needs. The EUnetHTA Core Model is composed of 9 domains, each including several topics. Each topic also includes different issues (ie, questions that should be considered for the evaluation of health technologies). Thus, the model is structured into 3 levels: *domain* (level 1), *topic* (level 2), and *issue* (level 3). The combination of a domain, topic, and issue is linked to an assessment element ID, which can be identiﬁed using a speciﬁc code for standardization purposes (B0001, B0002, etc).

The main EUnetHTA model domains include the following: (1) health and current use of the technology, (2) description and technical characteristics of the technology, (3) safety, (4) clinical effectiveness, (5) costs and economic evaluation, (6) ethical aspects, (7) organizational aspects, (8) patient and social aspects, and (9) legal aspects.

The ethical domain (level 1) in the EUnetHTA Core Model [52] includes 5 topics (level 2): “benefit-harm balance,” “autonomy,” “respect for people,” “justice and equity,” and “legislation.” Each of these topics includes several issues (level 3) [52].

In this study, 2 authors (CP and ASR) independently analyzed the 25 selected articles. First, they read the articles several times to improve familiarity with the ideas addressing the ethical aspects of RCSs. Then, in each publication (methods, results, and discussion sections), they identified segments of data that were relevant or captured an idea linked to the “ethical” domain of the model. A subsequent exploration of the coded data (sentences or set of statements) was performed to get a more precise classification at the topic level (level 2) and at the issue level (level 3). Then, the coding was performed using the HTA nomenclature. The 2 experts (CP and ASR) compared their results. In a few cases, the coding results showed a lack of consensus between the 2 coding authors, which was resolved through a subsequent discussion between them. Interrater correlation was not calculated.

A thematic analysis using the EUnetHTA framework for conducting a literature review has been described in other studies [56,57]. Furthermore, the use of EUnetHTA to perform an ethical analysis of health technologies has already been proposed [58]. The 25 selected articles were all coded using this methodology. Some authors have previously emphasized the possibility of overlapping issues between topics in the HTA analysis. They have suggested to assess the overlapping issues in the most relevant topic section [59].

This review was not registered, and a protocol for the review was not prepared.

## Results

Selected articles are presented in Multimedia Appendix 1 [3,12,18,31,32,37-51,60-64]. For each topic, we have presented our findings in terms of questions and recommendations according to the EUnetHTA Core Model, wherever possible.

### Ethical Issues and Recommendations for the Use of New Technologies

This section aims to summarize the ethical analysis performed regarding the use of RCSs with older adults and to provide recommendations for ethical use of these devices. Table 1 presents a synthetic summary of the elements presented in this section.

**Table 1:** Summary of the main ethical issues and recommendations on the use of robotic coaching solution in older adults following the Health Technology Assessment–inspired analysis.

Topic and ethical issues (European Network of Health Technology Assessment Core Model)			Ethical concerns		Recommendations
**Topic 1: benefit-harm balance**					
	What are the known and estimated benefits and harms for patients when implementing or not implementing the technology?	Risk of social isolation Risk of deception Bias of algorithms		To develop devices that promote human interaction To provide documentation and to explain in a clear and understandable way the functioning of the devices but also their benefits in a clear and understandable manner To explain the ins and out of the algorithms used in the device in detail	
	What are the benefits and harms of the technology for relatives, other patients, organizations, commercial entities, society, etc?	Tensions with proxy Ecological impact		To evaluate the impact of technological devices on users To evaluate the impact on the environment To encourage material recycling To develop energy-efficient technologies	
	Are there any unintended consequences of the technology and its application for patients?	Quickly evolving technology Unsuitability of technology		To offer user simplified training to the user To create devices adapted to the needs and preferences of end users (user-centered design)	
**Topic 2: autonomy**					
	Is the technology used for individuals who are especially vulnerable?	Informed consent		To ensure obtain consent from users before the use of a technological device To offer advance directives and a proxy to sign consent To ensure regular collection of consent and reminders of information about the technology	
	Does the implementation or use of the technology affect the patient’s capability and possibility to exercise autonomy?	Dependence on the technology Loss of freedom Creating a new source of authority		To continually reassess the trust placed in technological devices To regularly check that the system is adapted to the user and serves their interests	
**Topic 3: respect for persons**					
	Does the implementation or use of the technology affect human dignity?	Stigmatization		To promote positive information	
	Does the technology invade the sphere of privacy of the patient or user?	Privacy Security of data		Users must have control over the technology and know where their data will be stored The systems must also comply with the relevant data protection framework	
**Topic 4: justice and equity**					
	How does implementation or withdrawal of the technology affect the distribution of health care resources?	Societal pressure Digital divide Inequalities in resources		Ethical approval should be required obtained before technologies can be used To ensure access to all, open-source development is preferred to ensure access to all people To involve communities as much as possible	
	How are technologies with similar ethical issues treated in the health care system?	Replacement of professionals		Devices should only be used to enhance the work of caregivers, not to replace them Caregivers must have the opportunity to train on the use of these devices	
**Topic 5: legislation**					
	Can the use of the technology pose ethical challenges that have not been considered in the existing legislations and regulations?	Safety of devices Regulation of technology		To develop specific legal frameworks An emergency stop button is required in these devices An ethics committee must be created to help researchers develop ethical devices Researchers need to keep abreast of regulatory developments	

#### Topic 1: Benefit-Harm Balance

RCSs should be developed according to the principles of beneficence (ie, to promote the interest of users) and nonmaleficence (ie, to avoid inflicting harm) [39,60,64].

##### What Are the Known and Estimated Benefits and Harms for Patients When Implementing or Not Implementing the Technology?

###### Risk of Social Isolation

According to Sharkey and Sharkey [50], technological devices, when used appropriately, could benefit older adults by promoting social interaction and connection with their loved ones [4,31,40]. Broadbent et al [19] have discussed the potential of robots to reduce older adults’ social isolation. However, other authors reported the negative influence of the use of robotic devices on human contact [31,32,65]. The use of robots (eg, telepresence robots) to make some cost savings (eg, reducing travel costs and time spent on trips for family and professionals to visit older adults) would reduce face-to-face interactions [3,36,39,40]. Moreover, according to Körtner [47], the more people become accustomed to communicating with robots, the less they will be used to communicating with humans. The use of social robots could lead to a reduction of interactions with humans and thus to social isolation and emotional dependence [39]. However, the influence of technological devices, such as RCSs, on social isolation is still under debate, and the impact of technology would depend on the manner in which it is used.

To avoid exacerbating the users’ social isolation, Portacolone et al [38] advocate that social robots and similar technologies should be designed with the objective of fostering interactions with other humans, for instance, keeping users informed about the entertainment and socializing activities near their home, connecting them with their loved ones, and so on.

###### Risk of Deception

Another major risk for users is deception [39,64,66]. Portacolone et al [38] described 3 types of deception that people with neurocognitive disorders may face when interacting with social robotic systems but which may also apply to all users. The first type involves the user’s misconception of what is driving the technological device [51]. Users may be misled if they think that behind a medical chatbot, there is a real physician who communicates and reads their messages [44] or, alternatively, if they are not aware that, at some point, there are real humans guiding the technological device [38]. The second type refers to robotic devices programmed to express feelings or other types of affective communication, which may lead the user to believe that the system’s emotions are authentic. Related to this issue, Körtner [47] discussed how some older adults may fear that their social robot will forget them during their absence from home. The resemblance with the living in terms of affective behavior (eg, crying, laughing, or expressing concern) can make the user believe that there is a reciprocity between human and robot feelings [43]. The last type of deception is related to the inadequate interpretations that older adults may have regarding the nature of the robot, for example, thinking that an animal-shaped robot is a real animal or a pet [38]. Some current developments of social robots tend to make them resemble a living being, in terms of their verbal and nonverbal behaviors [34,60] or by highly anthropomorphizing their design [47], which may blur the boundary between the real and the artificial [45,60]. These design choices can also impact users’ dignity by infantilizing them as they are led to believe in something that is false [50].

However, according to some researchers [51,63,64], the notion of deception should be considered in terms of the gradation between what is morally acceptable and what is not. Deception would be morally acceptable when it aims to improve a person’s health or quality of life, for example, the use companion robots to calm a person experiencing behavioral disorders linked to dementia [51].

According to Danaher [43] and Vandemeulebroucke et al [40], to avoid deception, it is essential to be transparent to users about the design and operation of devices. As the information given to the participants is the basis for obtaining consent to use the technology, it is essential to offer them documents explaining how the device is built and its advantages and limitations in a clear manner adapted to the user’s knowledge and experience. It is also important to inform users on how to behave with technology [12]. Researchers should also answer users’ questions, pay attention to their feedback, and use it to improve the device and its documentation [60]. During experiments with RCSs, it is also important that researchers regularly remind participants of the nature of the technological device to reduce the risk of misinterpretation and to ensure that they still consent to participate in the study [38].

###### Biases of Algorithms

An autonomous device does not work without AI or algorithms that allow it to make decisions. However, these technologies are created by humans, and programming biases can be incorporated into them and lead to failures [44]. A technological device can, for instance, misread a situation and react accordingly, leading to a safety risk for the user [18]. Thus, it is essential that the researcher scrutinizes the algorithms used in RCSs before their implementation [44]. Fiske et al [44] also suggest providing the users with detailed explanations about the algorithms present in the technological device they are using.

##### What Are the Benefits and Harms of the Technology for Relatives, Other Patients, Organizations, Commercial Entities, Society, Etc?

At the society level, Boada et al [39] mentioned an ethical consideration related to the ecological impact of robotic devices in the current context of climate crisis and the lack of natural resources. The construction of RCSs requires raw materials, high energy consumption, and the management of their waste. Therefore, it is important for developers to design technologies that consume less energy and can be recycled.

##### Are There Any Unintended Consequences of the Technology and Its Application for Patients?

###### Technologies Evolving Very Quickly

For some older adults, technologies evolve very quickly, which makes it difficult for them to keep up with [62]. Denning et al [67] encourage designers to develop products that are intuitive to use or to offer users a simplified training. However, although some technologies are progressing quickly, technological limitations are still present, especially regarding social robotic systems, impacting their performance [68] and generating frustration among some users [69].

###### Unsuitability of Technology

The lack of experience with the technologies and the fact that the systems are not suitable to everyone can reduce the usability and acceptability of RCSs among older adults [3,60,62]. Frennert and Östlund [62] highlighted that some older adults were not confident in their ability to handle a robot because of previous complicated experience with technology. Peek et al [70] also reported that users had doubts about their ability to use the technology and feared that they would easily forget how to use it. They may also fear false alarms generated by monitoring technologies. For example, a person may decide to sit on the floor, but this behavior can be considered as a fall by the technology, and it could call for an ambulance to be sent to the person’s home in vain [70].

To promote acceptability and usability of RCSs, it is essential to develop them considering the capabilities, needs, and wishes of various users [31,47]. “User-centered design” approaches should be used for this purpose [71]. This methodology must be performed in a continuous manner to consider the development, new preferences, and experiences of the users. Technology assessment should also be conducted before deployment in ecological environments to improve the predictability of RCSs and decrease the risk of confusion and accidents [40,47].

#### Topic 2: Autonomy

According to Anderson and Kamphorst [42], the notion of autonomy implies the recognition of people, for instance, users of RCSs, as thinking individuals who have their own perspective on matters and are able to judge what is best for them.

##### Is the Technology Used for Individuals Who Are Especially Vulnerable?

Free and informed consent is a prerequisite for the involvement of an individual in research, regardless of the domain. This aspect is mentioned in numerous codes and declarations such as the Declaration of Helsinki (1964-2008) [72]. In the context of studies of the use of RCSs, this principle ensures that the person has freely chosen to use a device. However, some older adults, particularly those with cognitive disorders, may have difficulties in understanding and evaluating information related to RCSs and therefore in making appropriate choices [3]. Moreover, the person may not remember that the RCS is in their environment or how it works [38,44]. The question of how to ensure that the older adult has understood the purpose of RCS and that their choice of using the technology is based solely on their own decision and not that of a relative, caregiver, or institution has also been discussed [46].

Researchers in the field of RCS should adapt to the cognitive abilities of the populations they work with to facilitate communication and decision-making [46]. Thus, the observation of the person’s behavior is necessary to identify potential reservations regarding the use of RCSs. When the person is very vulnerable to respond, informed consent could be sought by proxy such as from children, spouse, or partner [46,64]. However, according to Diaz-Orueta et al [37], the final decision of using RCSs lies with the user. To prevent loss of capacity and to guard against any risk of inducement to participate, advance directives [46,64] or implementation of an advance power of attorney [46] can be proposed.

##### Does the Implementation or Use of the Technology Affect the Patient’s Capability and Possibility to Exercise Autonomy?

###### Dependence on the Technology

Although the main interest of RCSs for older adults is the maintenance of functional independence, it has been claimed that these devices could make people dependent on them. By replacing users in tasks that they can still perform, the use of RCSs could create new forms of vulnerability [3,31,39,41,51].

People could rely entirely on autonomous technological devices, such as RCSs, to guide their behaviors, goals, and actions [12,73]. A questioning of the authenticity of users’ actions has been mentioned by Anderson and Kamphorst [42]. Users might not feel responsible for the success of their actions if they feel they are completely driven by the guidance of the RCS. People could also develop emotional and psychological feelings toward the technology. This may have negative consequences for the individuals [38,49] and lead to new vulnerabilities [39].

###### Loss of Freedom

Another ethical issue relates to the conflict between the user’s safety, encouraged by the technology guidance, and a loss of freedom. The RCS could impose constraints on the user under the pretext that the user’s actions are not good for them [39,40,74]. Sharkey and Sharkey [50] explained that to promote home care, RCS could act as a supervisor (ie, programmed to ensure that no danger is present and, if there is a danger, to implement procedures to stop it and avoid it in the future). For instance, the RCS could prevent the person from eating fatty and high-caloric food because it is harmful to them. To protect users and ensure that they live in good health, individuals using RCSs could end up being deprived of certain actions or being under some type of “house arrest” [50].

One of the goals of using such RCSs is to support older adults’ independence; therefore, it is essential that developers and researchers in the field take measures to preserve the person’s autonomy [75]. Furthermore, RCS users must have the opportunity to evaluate and re-evaluate the role given to the device, to assess whether the system is reliable and whether it is serving their interests [12,42].

###### Creating a New Source of Authority

The use of RCSs could alter human relationships, for example, by creating tensions between older adults and their informal caregivers. Their use could also create some tensions with health care professionals by creating a new source of authority [12]. Monitoring older adults through RCSs can generate anger in the user, for example, when the device insists that the older adult should take a medication that they do not want to take [41,75].

#### Topic 3: Respect for Persons

##### Does the Implementation or Use of the Technology Affect Human Dignity?

Human dignity may be affected by the use of RCSs as these technologies may be perceived as “problem evocators” [41]. Some RCSs are used to compensate for impaired capacities. However, according to Körtner [47], their use can make older adults aware of their limitations and lead to negative feelings, anxiety, or exhaustion. RCS use can also lead to a form of stigmatization by making one’s own inabilities visible to others [3,70]. It is important to have positive communication regarding RCSs, to provide a less stigmatizing view of their use.

##### Does the Technology Invade the Sphere of Privacy of the Patient or User?

###### Privacy

To continue living at home, users are increasingly willing to tolerate intrusion in their privacy [70], but they are not always aware of when and how they are being monitored by RCSs [61]. Portacolone et al [38] provided the example of an animal-shaped companion robot, for which the older adults can signal that they no longer wish to interact with it by putting the robot to sleep. However, the animal-shaped robot can record data even when it is sleeping, but users are not always aware of this information. Forgetfulness and the lack of understanding of the device can lead to the risk of manipulation and coercion [44]. The person who is vulnerable may forget that they are being monitored and reveal personal information [50].

Technological devices, such as RCSs, must remain under the control of the users [47]. Users should have the ability to define when and where the device is used—when it collects data—to maintain their privacy, especially in intimate or private care settings.

###### Security of Data

According to Portacolone et al [38], remote monitoring technologies are usually controlled by third parties, sometimes even operating in another country, which can lead to cultural biases during the interaction between the older adult and the RCS. This context involves the risk that the person controlling the device (third party) takes advantage of the older adult’s vulnerability to steal their personal information or exposes the user to financial abuse [38]. Older adults are not always aware or vigilant about the sharing and use of data, which may be personal and sensitive [73]. Furthermore, RCSs can be connected to internet services that collect, store, and transfer these sensitive data [47] for commercial use [49,61].

In addition, the use of technologies connected to digital networks involves the risk of hacking and unauthorized surveillance [34,51], which can make people vulnerable [62]. Denning et al [67] found that home robots could not only be remotely located and identified but also hacked and controlled. First, users may have either preconceived and erroneous ideas about the capabilities of the device or a lack of knowledge to evaluate the safety, especially regarding data protection [3]. Second, users do not always configure their technological device correctly or update them [67].

Encryption or security systems must be put in place to protect users’ personal data captured by the devices at every stage: during collection, storage, transmission, and processing [3]. Researchers must also give particular attention to data security. In Europe, for instance, researchers and technology providers are required to comply with the General Data Protection Regulation [40,76]. Data collection must be performed legally or approved by the local relevant ethical committees.

To address data security challenges, 3 principles are recommended by Ienca et al [46] when developing technological devices: transparency, legitimate purpose, and proportionality. Transparency refers to the fact that the user knows that the system is collecting data and has consented to it. The user must also have precise information about when and what type of data are recorded and who has access to them [47]. Legitimate purpose refers to the notion that the monitoring and collection of data is performed for a specific purpose, (ie, in the best interest of the user or, if applicable, a relative who has consented to it). Finally, the principle of proportionality refers to the fact that the data collected are not disproportionate to the user’s needs.

#### Topic 4: Justice and Equity

The consequences of the technology implementation on the distribution of health care resources was discussed in the literature.

##### How Does Implementation or Withdrawal of the Technology Affect the Distribution of Health Care Resources?

###### Societal Pressure

Socioeconomic issues are also linked to the development and use of RCSs can also be raised. Individual freedom may be hindered by the “incentive” of certain stakeholders or authorities to enforce the use of RCSs [37]. The use of RCSs and similar systems may also lead to a lesser involvement of relatives, caregivers, and institutions that provide care to older adults and to the reduction of care costs; these perceived economic benefits may pressurize older adults to consent to use these devices [40,46]. It is also possible that older adults may have to agree to use the technological device to receive other health care benefits (eg, aids and subsidies) [42].

###### Digital Divide

Different opportunities to access RCSs can result in digital divide, defined by the Organisation for Economic Cooperation and Development [77] as a gap between those who have access to information and communication technologies and those who do not. This difference can create educational, economic, social, and even health-related disparities among citizens. Some citizens would be able to use these devices and, therefore, could benefit from their advantages, whereas others will not be able to use them and will not enjoy their benefits. The use of technologies in the health care context, through public or private institutions, should be subject to previous authorization by independent ethical committees to ensure that the use of these devices will not harm users in any way.

###### Inequalities in Resources

Questions about justice, equity, and equality among all citizens also arise [12,40,46]. RCSs have relatively high costs [64] and can generate additional expenses such as an internet subscription [3] that only a part of the population can afford, and this may be owing to the lack of research allowing to measure the cost-to-benefit ratio of these technologies on health [32]. It is important to ensure the access to RCSs among different living areas (ie, urban and rural). Therefore, involving municipalities and neighborhood associations seems an interesting way of raising awareness about the opportunities offered by RCSs for older adults and reaching a wider range of people.

To promote justice, equity, and fair distribution, Ienca et al [46] and Wangmo et al [64] recommend reducing the development costs of RCSs by promoting an open dissemination of source codes. In addition, RCSs should be distributed in priority to those in greatest need; therefore, measures to ensure access to RCSs under fair conditions should be established [51]. Joachim [78] also suggests to cover some of the costs of these health care–oriented technologies through health insurance.

Recommendations have been published by researchers to improve equality of access to technologies, such as using open-source software, providing priority access for individuals with low income, or relying on certain collective financing systems such as retirement or health insurance [46,51,78]. Discussions must be conducted among developers, legislators, and private and public organizations to identify viable financing solutions that allow for fair distribution of RCSs.

###### Replacement of Professionals

Researchers have also reported fears expressed by older adults and caregivers about how the use of technological devices could eliminate care-related jobs or replace humans [17,34,48,61]. There are also concerns about the use of these technological tools to reduce health care costs by decreasing the number of available health care resources and services, thereby exacerbating social inequalities [44]. The introduction of health-oriented RCSs requires adapting the contexts of care practices, which may threaten their quality [39]. Their incorporation into the care work environment can be difficult because the devices are automated and some care situations are unpredictable [17,62]. Furthermore, the gestion of certain tasks by technological devices requires a restructuring of the roles and responsibilities of caregivers [39]. Fiske et al [44] highlight that there are currently no recommendations or training to enable health care professionals to adopt RCSs, even though these professionals are increasingly confronted with technological devices in their practice.

The incorporation of RCSs must always be accompanied by a discussion with concerned care professionals regarding the advantages and limits of the technology. Professionals must also be supported in the use of these devices through effective training. Structured training and supervision will contribute to the development of a controlled framework of practice around the use of RCSs and thus avoid potential abuse [44]. Moreover, to encourage their use among professionals, it is essential to clearly define the role of RCSs as an additional resource for professionals and not a replacement of human care services [44].

#### Topic 5: Legislation

The ethical challenges linked to the lack of existing legislations and regulations dedicated to the use of the technology were discussed in the literature.

##### Can the Use of the Technology Pose Ethical Challenges That Have Not Been Considered in the Existing Legislations and Regulations?

###### Safety of Devices

The use of RCSs by older adults can result in damage and harm to their environment [79], especially when the device is still at the prototype stage [47]. Safety risks linked to the use of RCSs (eg, malfunctioning of the technology and incorrect decisions made by the coaching system) arise when they share a common space with humans and interact with them [39]. The following questions must be considered: Who is responsible in case of an accident, and who pays for the damages [39,40,48,62,80]? Is it the designer, the device, or the user himself? Currently, the civil code favors the cascade system (ie, first, the liability falls on the designer of the product; then, on the developer; and finally, on the user who has not followed the rules of use) [74]. However, the more the machine becomes autonomous, the less the existing legal frameworks can answer these questions [80]. This is a key legal issue regarding the implementation of RCSs in real settings because the person responsible for damage to the user or the environment may incur legal or even penal proceedings.

Damage and prejudice can also be caused by a failure to share authority [45,49,60]. Who between the human and the technological device holds the power to make decisions and control a functionality [81]? According to Grinbaum et al [45], it is important to specify the circumstances in which the human must take control over the technological device (RCS) and those in which the device should decide autonomously. According to Riek and Howard [49], it is preferable that in certain cases, the technological device, although autonomous, requires a human validation of its actions to keep the user in control of the device. In addition, Bensoussan and Puigmal [80] suggested the idea that technological devices must have an emergency stop button, so that the human can switch off the technology at any time.

###### Regulation of Technology

Currently, there is a gray area between the capabilities of RCSs, the reality of the field, and the regulations in force [38]. To accompany the researcher during the whole process of development and diffusion of RCSs, an ethical framework should be established [18,60]. Specifically, this can be in the form of an ethical code of conduct illustrating the expectations to all the employees of a company [18]. The researcher must regularly inform themselves about the ethics to be consistent with the evolution of the regulatory framework [60]. However, according to Nevejans [82], these ethical recommendations have no legal value and cannot protect humans from the damage caused by new technologies. Thus, it is necessary to think about a new legal framework to protect the users of RCSs [37].

## Discussion

### Summary

The use of technologies, such as RCSs, in the health care field has grown significantly in recent years [17,18]. RCSs are increasingly being used for older adults with the aim of promoting healthy behaviors, quality of life, and well-being. However, the use of RCSs also raises several ethical challenges regarding the cost-to-benefit balance of these new care practices, respect for the autonomy of users, respect for privacy, justice and equity linked to their access, or need for a suitable legal framework. Such challenges could be addressed by establishing relevant recommendations for the development and use of RCSs. Some guidelines regarding the use of robotic systems have been published [49,83]. Moreover, in April 2021, the European Commission unveiled the first legal framework about AI [84]. However, to the best of our knowledge, no recommendations have been proposed in this field directly linked to an analysis of the literature dealing specifically with these ethical issues and potential solutions to address them.

This narrative review identified 25 articles in which authors highlighted ethical issues and recommendations related to the use of RCSs and similar technologies. The use of the EUnetHTA Core Model for the analysis of these articles made it possible to classify the information retrieved in the publications according to 5 main ethical topics—“benefit-harm balance,” “autonomy,” “respect for persons,” “justice and equity,” and “legislation”—and to provide a detailed analysis of RCS-related ethical issues. Our review also aimed to identify recommendations for better development, diffusion, and use of RCSs by a population of older adults.

Technology devices, such as RCSs, are used with older adults to enable them to live independently; to enhance their quality of life and well-being; and, therefore, to cope with the increasing care demands for older populations. RCSs may be used to encourage a range of health-related goals: physical, cognitive, nutritional, social, and emotional domains. To be effective, RCSs must be able to motivate the user by providing highly personalized care programs [85,86]. However, studies have shown that not all potential target users are included in the development of these devices [37,87,88]. Therefore, RCSs design might fail to meet a wide range of users’ needs, capabilities, and wishes. Thus, it is essential to apply “user-centered design” approaches and involve target users with various sociodemographic characteristics and technology experience throughout the development process. A strong involvement of the intended users of these systems in their design process would also improve the quality of the information provided to potential users of RCSs regarding their operation, type of data collected, and potential benefits of the technology. In this way, the involvement of the users would improve the quality of the process of obtaining the consent required from older adults to use the technology.

Another ethical challenge related to the use of RCSs is the fact that their wide implementation for older adults’ care may affect the distribution of health care resources. For instance, it has been found that for some older adults and informal and formal caregivers, the use of RCSs could replace humans in many caregiving tasks, eventually leading to a suppression of jobs or to a degradation of the quality of health care services [17,34,48,61]. In this regard, the participation of a third person (professional, volunteer, or family member) as a “human coach” could be considered when implementing RCSs in the older adults’ environment. This “human coach” could help build a “chain of trust” by being an intermediary between the RCS and the user. On the one hand, the involvement of a real person in the use of the RCS could reduce the risk of replacement of human assistance by technological assistance. On the other hand, the “human coach” could help enhance the acceptability and usability of the device, while at the same time, reassuring the user and providing recommendations to the developers, so that the RCS is consistent with users’ needs and desires. However, the benefits of involving a “human coach” in the RCS service provision has yet to be evaluated by scientific studies.

According to some studies [3,39,41,51,65], the use of RCSs can have an impact on social relationships, reducing human contact and even altering social relationships by creating tension between older adults and their caregivers. Thus, it would be interesting to identify the repercussions and implications of these devices in older adults’ daily life and in the life of the members of their social environment through new studies. It also seems necessary to evaluate the organizational impact of the implementation of RCSs and to identify potential obstacles to their use in the care professionals’ work context.

Our analysis also confirmed that for RCSs to provide personalized health-related recommendations, the collection of sensitive data is necessary. Data collection in this context also raises several ethical issues. For instance, personal data can be exposed to hacking and misuse. Proper data management, anonymization, and encryption are essential to protect the personal data of RCS users [86]. In addition, researchers and developers in this field must evaluate RCSs before implementation to ensure that they do not cause physical or moral harm to users. Thus, it has been suggested that stakeholders refer to local and regional regulatory and safety standards to guide their development and use.

Finally, our analysis also discussed how legal and ethical frameworks regarding the use of RCSs need to be adapted to cope with the constant development of new technologies. So far, existing legal frameworks are not yet adequate to respond effectively to the question of liability in case of damage caused by RCSs, particularly because these devices are becoming increasingly autonomous [80]. The establishment of “operational ethics committees in digital sciences and technologies” could help in the development and conduct of projects in this area [60]. Guidelines should be established to identify the types of applications and technological devices that require regulatory review and approval [44]. Research projects and working groups involving users, researchers, and lawyers should be set up to further investigate the legal and ethical issues related to the use of RCSs.

Some countries and regions, such as Europe and Japan have initiated the work of structuring relevant legal and ethical frameworks; however, their orientations and measures may differ culturally [78]. Future studies in the area of RCSs could consider the influence of cultural and socioeconomic specificities of the contexts of experimentation (countries and regions) regarding the acceptance and use of RCSs by older adults and formal and informal caregivers and regarding the definition of ethical and legal frameworks governing their uses. Therefore, the use of validated and widely applied analysis frameworks, for example, the Western, Educated, Industrialized, Rich and Democratic framework [89], formulated to measure countries’ commonalities in their approaches to the interpretation of behavioral research findings (eg, regarding technology adoption) could be interesting. The Western, Educated, Industrialized, Rich and Democratic framework [89] could help not only to explore the differences among countries regarding the validation and adoption of new technologies for older adult care but also to seek greater cultural and demographic diversity in technology research.

This dimension of cross-cultural comparison has received particular attention in the framework of a current international research partnership between Europe and Japan, such as the EU-Japan Virtual Coach for Smart Ageing (e-VITA) project. This project aims to develop a cross-cultural RCS that can be tailored to the needs of healthy older adults to promote aging well. The e-VITA RCS will be made available to older adults in their homes, which raises many of the ethical questions discussed in this paper. Therefore, the study will require the researchers to set up procedures adapted not only to the users but also to the 2 cultures (European and Japanese), respecting the corresponding ethical and legal regulations. Thus, it would be interesting to perform an analysis of the ethical issues raised by users from different countries and cultures within the framework of the e-VITA project.

### Limitations

A narrative review of the literature was conducted to provide a nonexhaustive synthesis of the various ethical concerns and recommendations when using RCSs for older adults. This review has some limitations. Only articles in French and English were included. Some articles indicating ethical concerns or recommendations may not have been included when this information was not mentioned in the keywords or abstract.

### Conclusions

The use of RCSs in the context of health care, particularly with an older adult population, tends to show many benefits. RCSs have the potential to improve the quality of life of older adults and their independence. When used in an ethical and appropriate manner, RCSs can help improve older adults’ emotions and cognitive and physical abilities and promote social relationships. By helping older adults to continue living at home for as long as possible, the use of health-oriented RCSs could help to address some of the challenges resulting from demographic aging. However, the use of these new health care technologies involves some ethical concerns, with the most cited issues being not only the risk of accidents, lack of reliability, loss of control, risk of deception, and risk of social isolation but also the confidentiality of data and liability in case of safety problems.

Some recommendations have been made in the past regarding the use of social and assistive robotic technologies for older adults, such as considering the opinion of target users; collecting their consent; training the care professionals to use them; and ensuring proper data management, anonymization, and encryption. However, the integration of RCSs in current health practices and, particularly, in the private homes of older adults can be disruptive. It requires the establishment of scalable and adapted ethical and regulatory frameworks that follow the technology progress and the social and digital change of society Thus, studies are needed to identify new ethical concerns arising from the organizational impact of the implementation of RCSs in different contexts, especially in the homes of older adults. The influence of cultural and socioeconomic specificities of the contexts of experimentation (countries and regions) regarding the acceptance and use of RCSs by older adults and formal and informal caregivers is also an area of interest for future studies.

## Appendix

Multimedia Appendix 1 The selected relevant articles on ethical issues regarding the use of robotic coaching solutions for older adults.

## Abbreviations

AI: artificial intelligence
EUnetHTA: European Network of Health Technology Assessment
e-VITA: EU-Japan Virtual Coach for Smart Ageing
HTA: Health Technology Assessment
RCS: robotic coaching solution

## Data Availability

Data sharing is not applicable to this paper as no data sets were generated or analyzed during this review.

## References

[1] United Nations (2019). Croissant à un rythme plus lent, la population mondiale devrait atteindre 9,7 milliards d'habitants en 2050 et pourrait atteindre près de 11 milliards vers 2100 : Rapport de l'ONU. United Nations, Population Division.

[2] (2016). Rapport mondial sur le vieillissement et la santé. Organisation Mondiale de la Santé.

[3] Chung J, Demiris G, Thompson HJ (2016). Ethical considerations regarding the use of smart home technologies for older adults: an integrative review. Annu Rev Nurs Res.

[4] Ganesan B, Gowda T, Al-Jumaily A, Fong KN, Meena SK, Tong RK (2019). Ambient assisted living technologies for older adults with cognitive and physical impairments: a review. Eur Rev Med Pharmacol Sci.

[5] Palazzolo J, Baudu C, Quaderi A (2016). Psychothérapies du Sujet Âgé: Prise en Charge des Pathologies du Vieillissement. 2nd edition.

[6] Principaux repères de l'OMS sur la démence. World Health Organization.

[7] Guilbaud A, Mailliez A, Boulanger É (2020). Vieillissement: une approche globale, multidimensionnelle et préventive. Med Sci (Paris).

[8] op den Akker H, Klaassen R, op den Akker R, Jones VM, Hermens HJ (2013). Proceedings of the 26th IEEE International Symposium on Computer-Based Medical Systems.

[9] Görer B, Salah AA, Akın HL, Augusto JC, Wichert R, Collier R, Keyson D, Salah AA, Tan AH (2013). A robotic fitness coach for the elderly. Ambient Intelligence: Lecture Notes in Computer Science. Volume 8309.

[10] Lete N, Beristain A, García-Alonso A (2020). Survey on virtual coaching for older adults. Health Informatics J.

[11] Siewiorek D, Smailagic A, Dey A (2012). Architecture and applications of virtual coaches. Proc IEEE.

[12] Kamphorst BA (2017). E-coaching systems: what they are, and what they aren't. Pers Ubiquit Comput.

[13] Kyriazakos S, Schlieter H, Gand K, Caprino M, Corbo M, Tropea P, Judica E, Sterpi I, Busnatu S, Philipp P, Rovira J, Martínez A, Lange M, Gabilondo I, Del Pino R, Carlos Gomez-Esteban J, Pannese L, Bøttcher M, Lynggaard V (2020). A novel virtual coaching system based on personalized clinical pathways for rehabilitation of older adults-requirements and implementation plan of the vCare project. Front Digit Health.

[14] Wrobel J, Pino M, Wargnier P, Rigaud AS (2014). Robots et agents virtuels au service des personnes âgées : une revue de l’actualité en gérontechnologie. NPG Neurol Psychiatr Geriatr.

[15] Pérez PJ, Garcia-Zapirain B, Mendez-Zorrilla A (2015). Caregiver and social assistant robot for rehabilitation and coaching for the elderly. Technol Health Care.

[16] Fasola J, Mataric MJ (2013). A socially assistive robot exercise coach for the elderly. J Hum-Robot Interact.

[17] Cresswell K, Cunningham-Burley S, Sheikh A (2018). Health care robotics: qualitative exploration of key challenges and future directions. J Med Internet Res.

[18] Winfield AF, Jirotka M (2018). Ethical governance is essential to building trust in robotics and artificial intelligence systems. Philos Trans A Math Phys Eng Sci.

[19] Broadbent E, Stafford R, MacDonald B (2009). Acceptance of healthcare robots for the older population: review and future directions. Int J of Soc Robotics.

[20] Abou Allaban A, Wang M, Padır T (2020). A systematic review of robotics research in support of in-home care for older adults. Information.

[21] Cortellessa G, De Benedictis R, Fracasso F, Orlandini A, Umbrico A, Cesta A (2021). AI and robotics to help older adults: revisiting projects in search of lessons learned. Paladyn J Behav Robot.

[22] Lima MR, Wairagkar M, Gupta M, Rodriguez y Baena F, Barnaghi P, Sharp DJ, Vaidyanathan R (2022). Conversational affective social robots for ageing and dementia support. IEEE Trans Cogn Dev Syst.

[23] Sidner CL, Bickmore T, Nooraie B, Rich C, Ring L, Shayganfar M, Vardoulakis L (2018). Creating new technologies for companionable agents to support isolated older adults. ACM Trans Interact Intell Syst.

[24] Pepito JA, Locsin RC, Constantino RE (2019). Caring for older persons in a technologically advanced nursing future. Health.

[25] Pinheiro PR, Pinheiro PG, Filho RH, Barrozo JP, Rodrigues JJ, Pinheiro LI, Pereira ML (2020). Integration of the mobile robot and internet of things to monitor older people. IEEE Access.

[26] Chatterjee A, Gerdes MW, Martinez S (2019). eHealth initiatives for the promotion of healthy lifestyle and allied implementation difficulties. Proceedings of the 2019 International Conference on Wireless and Mobile Computing, Networking and Communications.

[27] Yousuf H, Reintjens R, Slipszenko E, Blok S, Somsen GA, Tulevski II, Hofstra L (2019). Effectiveness of web-based personalised e‑Coaching lifestyle interventions. Neth Heart J.

[28] Bevilacqua R, Casaccia S, Cortellessa G, Astell A, Lattanzio F, Corsonello A, D'Ascoli P, Paolini S, Di Rosa M, Rossi L, Maranesi E (2020). Coaching through technology: a systematic review into efficacy and effectiveness for the ageing population. Int J Environ Res Public Health.

[29] Justo R, Ben Letaifa L, Palmero C, Gonzalez-Fraile E, Torp Johansen A, Vázquez A, Cordasco G, Schlögl S, Fernández-Ruanova B, Silva M, Escalera S, deVelasco M, Tenorio-Laranga J, Esposito A, Korsnes M, Torres MI (2020). Analysis of the interaction between elderly people and a simulated virtual coach. J Ambient Intell Human Comput.

[30] Fiorini L, De Mul M, Fabbricotti I, Limosani R, Vitanza A, D'Onofrio G, Tsui M, Sancarlo D, Giuliani F, Greco A, Guiot D, Senges E, Cavallo F (2021). Assistive robots to improve the independent living of older persons: results from a needs study. Disabil Rehabil Assist Technol.

[31] Sriram V, Jenkinson C, Peters M (2019). Informal carers' experience of assistive technology use in dementia care at home: a systematic review. BMC Geriatr.

[32] Pilotto A, Boi R, Petermans J (2018). Technology in geriatrics. Age Ageing.

[33] Koceska N, Koceski S, Beomonte Zobel P, Trajkovik V, Garcia N (2019). A telemedicine robot system for assisted and independent living. Sensors (Basel).

[34] Broadbent E (2017). Interactions with robots: the truths we reveal about ourselves. Annu Rev Psychol.

[35] Suwa S, Tsujimura M, Ide H, Kodate N, Ishimaru M, Shimamura A, Yu W (2020). Home-care professionals’ ethical perceptions of the development and use of home-care robots for older adults in Japan. Int J Hum Comput Interact.

[36] Nordgren A (2018). How to respond to resistiveness towards assistive technologies among persons with dementia. Med Health Care Philos.

[37] Diaz-Orueta U, Hopper L, Konstantinidis E (2020). Shaping technologies for older adults with and without dementia: reflections on ethics and preferences. Health Informatics J.

[38] Portacolone E, Halpern J, Luxenberg J, Harrison KL, Covinsky KE (2020). Ethical issues raised by the introduction of artificial companions to older adults with cognitive impairment: a call for interdisciplinary collaborations. J Alzheimers Dis.

[39] Boada JP, Maestre BR, Genís CT (2021). The ethical issues of social assistive robotics: a critical literature review. Technol Soc.

[40] Vandemeulebroucke T, Dierckx de Casterlé B, Gastmans C (2018). The use of care robots in aged care: a systematic review of argument-based ethics literature. Arch Gerontol Geriatr.

[41] Zafrani O, Nimrod G (2019). Towards a holistic approach to studying human-robot interaction in later life. Gerontologist.

[42] Anderson J, Kamphorst B (2014). Ethics of e-coaching: implications of employing pervasive computing to promote healthy and sustainable lifestyles. Proceedings of the 2014 IEEE International Conference on Pervasive Computing and Communication Workshops.

[43] Danaher J (2020). Robot betrayal: a guide to the ethics of robotic deception. Ethics Inf Technol.

[44] Fiske A, Henningsen P, Buyx A (2019). Your robot therapist will see you now: ethical implications of embodied artificial intelligence in psychiatry, psychology, and psychotherapy. J Med Internet Res.

[45] Grinbaum A, Chatila R, Devillers L, Ganascia JG, Tessier C, Dauchet M (2017). Ethics in robotics research: CERNA mission and context. IEEE Robot Automat Mag.

[46] Ienca M, Jotterand F, Vică C, Elger B (2016). Social and assistive robotics in dementia care: ethical recommendations for research and practice. Int J of Soc Robotics.

[47] Körtner T (2016). Ethical challenges in the use of social service robots for elderly people. Z Gerontol Geriatr.

[48] Operto F (2011). Ethics in advanced robotics. IEEE Robot Automat Mag.

[49] Riek LD, Howard DA (2014). A Code of Ethics for the Human-Robot Interaction Profession. Proceedings of 2014 Conference on We Robot.

[50] Sharkey A, Sharkey N (2010). Granny and the robots: ethical issues in robot care for the elderly. Ethics Inf Technol.

[51] Yew GC (2021). Trust in and ethical design of carebots: the case for ethics of care. Int J Soc Robot.

[52] HTA Core Model ® version 3. EUnetHTA.

[53] (2015). Health technology assessment process: fundamentals. EUPATI Toolbox.

[54] Tuli TB, Terefe TO, Rashid MM (2021). Telepresence mobile robots design and control for social interaction. Int J Soc Robot.

[55] Green BN, Johnson CD, Adams A (2006). Writing narrative literature reviews for peer-reviewed journals: secrets of the trade. J Chiropr Med.

[56] Isabet B, Pino M, Lewis M, Benveniste S, Rigaud AS (2021). Social telepresence robots: a narrative review of experiments involving older adults before and during the COVID-19 pandemic. Int J Environ Res Public Health.

[57] Naudé B, Rigaud AS, Pino M (2021). Video calls for older adults: a narrative review of experiments involving older adults in elderly care institutions. Front Public Health.

[58] Saarni S, Hofmann B, Lampe K, Lühmann D, Mäkelä M, Velasco-Garrido M, Autti-Rämö I (2008). Ethical analysis to improve decision-making on health technologies. Bull World Health Organ.

[59] Lampe K, Mäkelä M, Garrido MV, Anttila H, Autti-Rämö I, Hicks NJ, Hofmann B, Koivisto J, Kunz R, Kärki P, Malmivaara A, Meiesaar K, Reiman-Möttönen P, Norderhaug I, Pasternack I, Ruano-Ravina A, Räsänen P, Saalasti-Koskinen U, Saarni SI, Walin L, Kristensen FB (2009). The HTA core model: a novel method for producing and reporting health technology assessments. Int J Technol Assess Health Care.

[60] Collectif C (2014). Éthique de la recherche en robotique. ALLISTENE.

[61] Tisseron S, Tisseron S, Tordo F (2018). Introduction. Robots, de Nouveaux Partenaires de Soins Psychiques.

[62] Frennert S, Östlund B (2018). How do older people think and feel about robots in health- and elderly care?. Proceedings of the 2018 INBOTS Conference on Inclusive Robotics for a Better Society.

[63] van Maris A, Zook N, Caleb-Solly P, Studley M, Winfield A, Dogramadzi S (2020). Designing ethical social robots-a longitudinal field study with older adults. Front Robot AI.

[64] Wangmo T, Lipps M, Kressig RW, Ienca M (2019). Ethical concerns with the use of intelligent assistive technology: findings from a qualitative study with professional stakeholders. BMC Med Ethics.

[65] Portet F, Vacher M, Rossato S (2012). Les technologies de la parole et du TALN pour l’assistance à domicile des personnes âgées : un rapide tour d’horizon (quick tour of NLP and speech technologies for ambient assisted living). Proceedings of the 2012 Workshop on Interactions Langagières pour personnes Agées Dans les habitats Intelligents.

[66] Bradwell HL, Winnington R, Thill S, Jones RB (2020). Ethical perceptions towards real-world use of companion robots with older people and people with dementia: survey opinions among younger adults. BMC Geriatr.

[67] Denning T, Matuszek C, Koscher K, Smith JR, Kohno T (2009). A spotlight on security and privacy risks with future household robots: attacks and lessons. Proceedings of the 11th international conference on Ubiquitous computing.

[68] Papadopoulos I, Koulouglioti C, Lazzarino R, Ali S (2020). Enablers and barriers to the implementation of socially assistive humanoid robots in health and social care: a systematic review. BMJ Open.

[69] Pripfl J, Kortner T, Batko-Klein D (2016). Results of a real world trial with a mobile social service robot for older adults. Proceedings of the 11th ACM/IEEE International Conference on Human-Robot Interaction.

[70] Peek ST, Wouters EJ, van Hoof J, Luijkx KG, Boeije HR, Vrijhoef HJ (2014). Factors influencing acceptance of technology for aging in place: a systematic review. Int J Med Inform.

[71] Lespinet-Najib V, Roche A, Chibaudel Q (2017). Santé et handicap: d'une conception centrée «utilisateur» à la conception universelle. Ann Mines Réal Ind.

[72] Déclaration d’Helsinki de l’AMM – Principes éthiques applicables à la recherche médicale impliquant des êtres humains. L'Association Médicale Mondiale.

[73] Adams S, Niezen M (2016). Digital ‘solutions’ to unhealthy lifestyle ‘problems’: the construction of social and personal risks in the development of eCoaches. Health Risk Soc.

[74] Saerens P (2020). Le droit des robots, un droit de l’homme en devenir ?. Commun Technol Dév.

[75] Jenkins S, Draper H (2015). Care, monitoring, and companionship: views on care robots from older people and their carers. Int J of Soc Robotics.

[76] (2016). Règlement (UE) 2016/679 du parlement européen et du Conseil du 27 avril 2016 relatif à la protection des personnes physiques à l'égard du traitement des données à caractère personnel et à la libre circulation de ces données, et abrogeant la directive 95/46/CE (règlement général sur la protection des données) (Texte présentant de l'intérêt pour l'EEE). Le Parlement Européen Et Le Conseil De L'union Européenne.

[77] (2001). Understanding the digital divide. OECD Digital Economy Papers.

[78] Joachim C (2020). Silver economy, robotique et droit - Comparaison franco-japonaise. Centre d’études et de Coopération Juridique Interdisciplinaire – Université de Poitier.

[79] Kernaghan K (2014). The rights and wrongs of robotics: ethics and robots in public organizations. Can Public Adm.

[80] Bensoussan A, Puigmal L (2017). Le droit des robots ? Quelle est l'autonomie de décision d'une machine ? Quelle protection mérite-t-elle ?. Arch Philos Droit.

[81] Tessier C, Lawless WF, Mittu R, Sofge D, Russell S (2017). Robots autonomy: some technical issues. Autonomy and Artificial Intelligence: A Threat or Savior?.

[82] Nevejans N (2017). Comment protéger l'homme face aux robots ?. Arch Phil Droit.

[83] Tambornino L, Lanzerath D, Rodrigues R, Wright D (2019). SIENNA D4.3: survey of REC approaches and codes for artificial intelligence and robotics. Zenodo.

[84] Ethics guidelines for trustworthy AI. Shaping Europe's Digital Future.

[85] Kamali ME, Angelini L, Caon M, Carrino F, Rocke C, Guye S, Rizzo G, Mastropietro A, Sykora M, Elayan S, Kniestedt I, Ziylan C, Lettieri E, Khaled OA, Mugellini E (2020). Virtual coaches for older adults’ wellbeing: a systematic review. IEEE Access.

[86] Banos O, Nugent C (2018). E-coaching for health. Computer.

[87] Gustafson DH, McTavish F, Gustafson DH, Mahoney JE, Johnson RA, Lee JD, Quanbeck A, Atwood AK, Isham A, Veeramani R, Clemson L, Shah D (2015). The effect of an information and communication technology (ICT) on older adults’ quality of life: study protocol for a randomized control trial. Trials.

[88] Sakaguchi-Tang DK, Cunningham JL, Roldan W, Yip J, Kientz JA (2021). Co-design with older adults: examining and reflecting on collaboration with aging communities. Proc ACM Hum Comput Interact.

[89] Henrich J, Heine SJ, Norenzayan A (2010). The weirdest people in the world?. Behav Brain Sci.

[90] EU-Japan virtual coach for smart ageing. e-VITA Consortium.

