# Supplementary material for: Identification of Ethical Issues and Practice Recommendations Regarding the Use of Robotic Coaching Solutions for Older Adults: Narrative Review
Source: J Med Internet Res. 2024 Jun 18;26:e48126. doi: 10.2196/48126 (PMC11220435; doi:10.2196/48126)
Supplement: Multimedia Appendix 1 [file jmir_v26i1e48126_app1.docx]

Table (Multimedia Appendix): The selected relevant articles on ethical issues regarding the use of robotic coaching solutions (RCSs) for older adults.

| Type of study | Study | Type of technology | Related ethical HTA^a^ topics | Principal Findings |
| --- | --- | --- | --- | --- |
| Theoretical | Anderson and Kamphorst [42], 2014 | e-Coaching | - Autonomy - Justice and equity | - e-Coaching supports individuals to maintain healthy and sustainable lifestyle. Ethical concerns were reported about self-regulation, privacy, autonomy, liberty, fairness, equality, responsibility, and authenticity |
| Theoretical | Cerna collective [60], 2014 | Robots | - Benefit-harm balance - Respect for persons - Legislation | - Recommendations for the use of robots (setting up an ethical committee, need for documentation, etc) |
| Theoretical | Danaher [43], 2020 | Robots | - Respect for persons | - Analysis of the risk of robot use and deception |
| Theoretical | Diaz-Orueta et al [37], 2020 | Assistive technologies | - Benefit-harm balance - Respect for persons - Justice and equity - Legislation | - Information about factors influencing technology adoption, role of autonomy and environmental control, challenges faced by older adults when using technology, and formulation of recommendations |
| Theoretical | Fiske et al [44], 2019 | Virtual agent AI^b^ agents, and robot therapy | - Benefit-harm balance - Respect for persons - Justice and equity - Legislation | - Significant benefits of AI applications in mental health and ethical considerations (risk assessment, referral, supervision, respect and autonomy, role of nonhuman therapy, transparency of algorithms, and long-term effects) |
| Theoretical | Grinbaum et al [45], 2018 | Robots | - Respect for persons - Legislation | - Privacy concerns, legal aspects, and dual use Recommendations about autonomy and decision-making abilities, life imitation, affective and social interaction, robot-assisted therapy, and human-robotic augmentation |
| Theoretical | Ienca et al [46], 2016 | Robots and intelligent assistive technologies | - Respect for persons - Justice and equity | - Benefits of using technology and ethical concerns (societal dimension and information deficit, informed consent, privacy and data security, safety, beneficence, nonmaleficence, autonomy, justice, equity, and fair distribution) |
| Theoretical | Kamphorst [12], 2017 | e-Coaching | - Benefit-harm balance - Autonomy - Respect for persons - Justice and equity | - e-Coaching has the potential to provide highly personalized assistance in a wide range of areas but raises several practical and ethical issues regarding privacy and personal autonomy |
| Theoretical | Körtner [47], 2016 | Robots and assistive technology | - Benefit-harm balance - Respect for persons - Legislation | - Benefits of technology for older adults; identification of ethical questions regarding deception, dignity, isolation, privacy, safety, and vulnerability; and recommendations to address these ethical issues |
| Theoretical | Operto [48], 2011 | Robots | - Justice and equity - Legislation | - The use of robots involves ethical reflection about human dignity and privacy, preservation of human and transhuman identity, liability issues, psychological effects, and cost-benefit analysis |
| Theoretical | Pilotto et al [32], 2018 | assistive technology, and robotics | - Benefit-harm balance - Justice and equity | - Benefits of technology on safety, communication and quality of life, and mobility and rehabilitation Ethical concerns about psychosocial and ethical issues and cost and fear of losing human interaction |
| Theoretical | Riek and Howard [49], 2014 | Robots and human-robot interaction | - Benefit-harm balance - Respect for persons - Legislation | - With the development of robots, new technical, ethical, and legal problems appear, for example, regarding the sharing of authority, human autonomy, lack of diversity, and respect for privacy |
| Theoretical | Sharkey and Sharkey [50], 2012 | Robots and assistive technology | - Benefit-harm balance - Autonomy - Respect for persons | - The use of robots has some benefits, but it also raises ethical concerns about 6 main issues: potential reduction of human contact, increased feelings of objectification and loss of control, loss of privacy, loss of personal freedom, deception and infantilization, and the circumstances under which older adults should be allowed to control robots |
| Theoretical | Tisseron [61], 2018 | Robots | - Benefit-harm balance - Respect for persons - Justice and equity | - Discussion of the advantages of using robots and identification of 3 main risks related to their use: privacy, deception, and relationship |
| Theoretical | Winfield and Jirotka [18], 2018 | Robots, AI, and intelligent autonomous systems | - Legislation | - Discussion about the benefits of using technology and concerns about loss of human contact, safety, authority, and job loss - Some recommendations were provided |
| Theoretical | Yew [51], 2021 | Care robot and AI | - Autonomy - Respect for persons - Justice and equity | - Challenges related to the use of care robots including deception, overdependence, and overattachment to care robots; obtaining informed consent for the use of care robots; and the privacy of users |
| Research paper | Frennert and Östlund [62], 2018 | Interactive robots | - Benefit-harm balance - Justice and equity - Legislation | - Potential for use of robots in health and older adult care, but some concerns (safety, reliability, vulnerability, and dependency) were also reported |
| Research paper | Portacolone et al [38], 2020 | Artificial companions | - Benefit-harm balance - Respect for persons - Legislation | - It is a cost-effective way to provide support Ethical issues include deception, surveillance and monitoring, informed consent, and social isolation |
| Research paper | van Maris et al [63], 2020 | Robots | - Respect for persons | - The use of robots may involve some emotional deception and emotional attachment |
| Research paper | Wangmo et al [64], 2019 | Intelligent assistive technology | - Autonomy - Respect for persons - Justice and equity | - Ethical concerns about informed consent, advance directive, deception, data access, data sharing, affordability, distributive justice, and human contact |
| Systematic review (56 papers) | Boada et al [39], 2021 | Assistive robot, care robot, social robot, human-robot interaction, and AI | - Benefit-harm balance - Autonomy - Respect for persons - Justice and equity - Legislation | - Ethical concerns regarding 3 main themes: well-being (eg, privacy, deception, autonomy, loss of human contact, and safety), care (eg, legitimacy, quality of practice, and humane moral practices), and justice (eg, distributive justice, accountability, social equality, robot decision-making, and ecological sustainability) |
| Systematic review (16 papers) | Chung et al [3], 2016 | Smart home technologies | - Benefit-harm balance - Autonomy - Respect for persons - Justice and equity | - Ethical concerns about privacy, informed consent, autonomy, obstructiveness, equity of access, reduced human contact, and ease of use |
| Systematic review (56 papers) | Sriram et al [31], 2019 | Assistive technologies | - Benefit-harm balance - Autonomy | - Assistive technologies have benefits and negative aspects regarding relationships, freedom, and autonomy |
| Systematic review (28 papers) | Vandemeulebroucke et al [40], 2018 | Robots | - Benefit-harm balance - Autonomy - Respect for persons - Justice and equity | - Ethical analysis can be conducted using 4 ethical approaches: deontological approach, principalist approach, goal list approach, and ethical approach to care |
| Systematic review (65 papers) | Zafrani and Nimrod [41], 2019 | Robots | - Benefit-harm balance - Autonomy - Respect for persons | - Benefits of the use of robotics and concerns about psychological risks, safety and reliability, and technological capabilities |

^a^HTA: Health Technology Assessment.

^b^AI: artificial intelligence.
